# Chronic Inhibition of STAT3/STAT5 in Treatment-Resistant Human Breast Cancer Cell Subtypes: Convergence on the ROS/SUMO Pathway and Its Effects on xCT Expression and System xc- Activity

**DOI:** 10.1371/journal.pone.0161202

**Published:** 2016-08-11

**Authors:** Katja Linher-Melville, Mina G. Nashed, Robert G. Ungard, Sina Haftchenary, David A. Rosa, Patrick T. Gunning, Gurmit Singh

**Affiliations:** 1 Department of Pathology and Molecular Medicine, McMaster University, Hamilton, Ontario, L8S 4L8, Canada; 2 Department of Chemical and Physical Sciences, University of Toronto Mississauga, Mississauga, Ontario, L5L 1C6, Canada; University of South Alabama, UNITED STATES

## Abstract

Pharmacologically targeting activated STAT3 and/or STAT5 has been an active area of cancer research. The cystine/glutamate antiporter, system x_c_^-^, contributes to redox balance and export of intracellularly produced glutamate in response to up-regulated glutaminolysis in cancer cells. We have previously shown that blocking STAT3/5 using the small molecule inhibitor, SH-4-54, which targets the SH2 domains of both proteins, increases xCT expression, thereby increasing system x_c_^-^ activity in human breast cancer cells. The current investigation demonstrates that chronic SH-4-54 administration, followed by clonal selection of treatment-resistant MDA-MB-231 and T47D breast cancer cells, elicits distinct subtype-dependent effects. xCT mRNA and protein levels, glutamate release, and cystine uptake are decreased relative to untreated passage-matched controls in triple-negative MDA-MB-231 cells, with the inverse occurring in estrogen-responsive T47D cells. This “ying-yang” effect is linked with a shifted balance between the phosphorylation status of STAT3 and STAT5, intracellular ROS levels, and STAT5 SUMOylation/de-SUMOylation. STAT5 emerged as a definitive negative regulator of xCT at the transcriptional level, while STAT3 activation is coupled with increased system x_c_^-^ activity. We propose that careful classification of a patient’s breast cancer subtype is central to effectively targeting STAT3/5 as a therapeutic means of treating breast cancer, particularly given that xCT is emerging as an important biomarker of aggressive cancers.

## Introduction

Aggressive cancer cells adapt to increased levels of reactive oxygen species (ROS) that accompany their dysregulated metabolism by up-regulating the activity of the plasma membrane antiporter, system x_c_^-^, which releases glutamate in exchange for cystine taken up from the extracellular environment. Imported cystine is essential to cancer cells, as it is intracellularly reduced to cysteine for the synthesis of glutathione (GSH), an antioxidant molecule that serves as one of the main mechanisms by which cancer cells effectively maintain redox balance (reviewed in [1]). System x_c_^-^ consists of the *xCT* (*SLC7A11*) gene product linked at the plasma membrane to the 4F2 heavy chain (4F2hc, CD98) [2]. In cancer cells, heterodimers of xCT-4F2hc may also complex with a variant of the CD44 cell adhesion molecule (CD44v) [3]. Of these components, xCT most prominently influences the function of system x_c_^-^, with high expression levels correlating with higher antiporter activity [2]. It has been suggested that insufficient xCT expression may sensitize cancer cells to ROS-mediated damage [3,4] and potentially curb their rapid proliferation [5] and metastatic potential [6]. It has also been speculated that high levels of xCT may be an indicator of chemo- and radiation therapy resistance [7], making it a target for novel drug development.

Signal transducer and activator of transcription (STAT) proteins, particularly STAT3 and STAT5, are constitutively activated in a large number of human cancers, where their sustained activity contributes to dysregulated proliferation, apoptosis, angiogenesis, and immune surveillance (reviewed in [8]). Therefore, these particular STATs have emerged as pharmacological targets for cancer therapy, and the development of novel STAT3/5 inhibitors is of considerable clinical interest for treating breast cancer, glioma, and leukemia [9–11]. In order to both positively and negatively regulate transcription, STATs are activated, homo- or heterodimerize, translocate to the nucleus, and bind to their recognition sites in target gene promoter regions, also potentially interacting with other transcription factors to modulate gene expression [12–14]. By regulating the expression of numerous target genes, including, for example, *MYC* [15,16], *BCL-2*, *BCL-XL*, cyclin D1 (*CCND1*), cyclin D2 (*CCND2*), p21WAF/Cip1 (*CDKN1A*), and p27kip (*CDKN1B*) [17,18], STAT family members dynamically control complex intracellular mechanisms. It has also been shown that STAT3 and STAT5 may oppose each other’s actions, producing reciprocal effects at target gene loci [19,20].

Numerous extracellular ligands, such as interleukin 6 and prolactin, induce canonical STAT3 and STAT5 singling cascades in relevant cell types [21,22]. ROS also modulate STAT activation in normal and cancer cells, and in turn, STAT3 and STAT5 modulate ROS production (reviewed in [23]). This latter effect may be due to their more recently described ability to translocate to the mitochondria, where they modulate cellular respiration and metabolism [24]. Given that system x_c_^-^ is intricately involved in redox homeostasis, we previously examined whether representative STAT family members regulate xCT expression, demonstrating by ChIP analysis that STAT3 and STAT5A are able to directly bind to the xCT promoter in MDA-MB-231 human breast cancer cells [25]. Our findings are computationally supported by several lines of evidence: xCT is listed as a STAT5A target gene based on published ChIP-chip, ChIP-seq, and other transcription factor binding site profiling studies assembled through the Ma'ayan Laboratory of Computational Systems Biology on-line database, Harmonizome (http://amp.pharm.mssm.edu/Harmonizome/dataset/CHEA+Transcription+Factor+Targets) [26], microarray studies indicate a link between STATs and changes in xCT mRNA levels [27,28], and PathwayNet predicts a transcriptional connection between xCT and STAT5A (http://pathwaynet.princeton.edu/predictions/geneset/?network=human-transcriptional-regulation&geneset=21547%2C13375). We have also shown that acutely blocking STAT3 and STAT5 phosphorylation using SH-4-54, a novel, structurally unique small molecule inhibitor that effectively targets interactions with the pTyr-SH2 domain to block both STAT3 and 5 dimerization and subsequent DNA-binding, also providing a promising means of targeting brain cancer stem cells [10], significantly up-regulates xCT expression and system x_c_^-^ activity in several human breast cancer cell lines [25]. This effect was coupled with increased ROS production [25] and potential NRF2 pathway activation, which has been computationally linked with STAT-mediated signaling through the JAK pathway via the NRF2-ome [29]. Interestingly, we observed that treating cells with a commercially available STAT5 inhibitor that targets the SH2 domain of STAT5 [30], inhibiting IFNα-stimulated STAT5 tyrosine phosphorylation without affecting STAT3 in human Burkitt’s lymphoma Daudi cells, increased xCT mRNA and protein levels without affecting intracellular ROS levels [31], suggesting that STAT5 could directly affect transcription at the xCT gene locus by acting as a repressor.

The current investigation examined the chronic effects of blocking STAT3/5 signaling in triple-negative MDA-MB-231 and estrogen receptor alpha (ERα)-positive T47D cells. Resistant clones representing sub-lines derived from both parental passage-matched wild-type breast cancer cell subtypes were selected based on their survival in response to chronic SH-4-54 treatment. Only a small number of individual treatment-resistant clones were obtained, confirming the overall potency of SH-4-54 and the importance of these STAT family members in breast cancer cells. SH-4-54-resistant clones were characterized based on their STAT3/5 activation profile (phospho-STATs relative to total protein), as well as changes in xCT mRNA levels, which were definitively confirmed by RNA-sequencing (NextGeneration analysis), as well as xCT protein levels and system x_c_^-^ activity. In addition, the stability of genotypic changes in the absence of further SH-4-54 treatment was assessed *in vivo* using murine xenografts. The overall goals of the current investigation were (1) to determine a potential mechanism by which blocking the activity of STAT3 and STAT5 affects system x_c_^-^, given the dynamic involvement of these particular transcription factors with mitochondrial function, redox balance, and the regulation of other key factors associated with cellular metabolism, which are all processes potentially interconnected with xCT expression, and (2) how these changes ultimately affect the genetic profile of different cancer cell types. Findings reported here may be of therapeutic interest for clinically applying STAT3/5 inhibitors to target cancers in which xCT expression is up-regulated, including gliomas and aggressive breast cancers.

## Materials and Methods

### Cell Lines, Culture, and Production of SH-4-54-Resistant Cell Lines

Both human cell lines were utilized in accordance with institutional biosafety guidelines. MDA-MB-231 and T47D human breast cancer cells lines were cultured according to the *in vitro* culture specifications outlined by ATCC. For clonal selection, cells were plated at several different densities into 10-cm dishes in either DMEM or RPMI supplemented with 10% fetal bovine serum to support the optimal growth of MDA-MB-231 or T47D cells, respectively. Media was changed every 2–3 days to administer SH-4-54 from a freshly thawed aliquot. After one or two months of continuous drug selection for T47D or MDA-MB-231 cells, respectively, individual clones were isolated by “picking” them using sterile cloning discs (Scienceware) presoaked in trypsin-EDTA. Each individual clone was transferred into one well of a 48-well plate and cultured to confluence in the presence of SH-4-54 prior to reseeding into a larger well format. For experiments, cells were plated into 6-well tissue culture-treated plates at 2.5x10^5^ cells/well 24 hours prior to manipulation. Untreated parental MDA-MB-231 or T47D cells, referred to as wild-type counterparts, were passaged in parallel. All cells were determined to be mycoplasma-free. Cell viability was assessed using trypan blue exclusion during cell count determination.

### Drugs

SH-4-54, a novel small molecule STAT3/5 inhibitor [10], was reconstituted in DMSO at a 25 mM stock. Individual aliquots were stored at -20°C, and cells were treated with vehicle or an appropriate concentration of drug (initially at 10 μM, followed by a 5 μM maintenance dose). Recombinant human prolactin (Cedarlane) was used at 100 ng/ml. Capsazepine (Cayman Chemical), paclitaxel (Sigma-Aldrich), and bleomycin (Sigma-Aldrich) were reconstituted in DMSO and used at final concentrations corresponding to 25 μM, 0.1 nM, and 1500 mU, respectively.

### Western Blotting

25–50 μg of each total cell lysate was loaded onto 10% polyacrylamide gels, which were subjected to SDS-PAGE electrophoresis coupled with immunoblotting as described previously [32]. Following chemiluminescent signal detection, stripped PVDF membranes were re-probed with anti-β-actin (13E5, #4970S; Cell Signaling Technology) or anti-calnexin (H-70, sc-11397; Santa Cruz Biotechnology) antibody. Other antibodies used for the current study included xCT (Novus Biologicals), phospho-STAT3 (Tyr705, D3A7; #9145), total STAT3 (79D7, #4904), phospho-STAT5 (Tyr694; #9351), and total STAT5 (#9363) (all from Cell Signaling Technology). Anti-rabbit or anti-mouse IgG horseradish peroxidase secondary antibodies were obtained from Cell Signaling Technology.

### Detection of SUMOylated Proteins by Immunoprecipitation

To detect SUMOylated proteins, cell extracts were prepared using lysis buffer supplemented with protease inhibitors plus N-ethylmaleimide (Pierce, Thermo Fisher Scientific) following the detailed protocol of Park-Sarge and Sarge (2009) [33]. Immunoprecipitation reactions (100 μg of total protein/1 reaction) were carried out with either normal rabbit IgG (DA1E; #3900, Cell Signaling Technology) as a negative control or total STAT5 antibody (sc-1081X, Santa Cruz Biotechnology). Immunocomplexes were isolated using protein A magnetic beads (#8687, Cell Signaling Technology) followed by standard Western blotting with either total STAT5, SUMO-1 (C9H1; #4940, Cell Signaling Technology), SUMO-2/3 (18H8; #4971, Cell Signaling Technology), or calnexin antibody as a negative control. Non-immunoprecipitated input was run in parallel on the same blots as a positive control.

### Luciferase Assay

Luciferase assays were carried out as described previously using the full-length human xCT promoter construct [31].

### Radiolabeled ^14^C-Cystine Uptake

Uptake of ^14^C-cystine (0.5 μCi/mL; Perkin Elmer) was determined as described previously [31]. Each lysate from cells plated 24 hours prior to performing the assay was run in duplicate for at least 3 independent experiments. Scintillation counts per minute were normalized to total protein, which was determined using the Bradford assay.

### Glutamate Assay

The level of glutamate released into the extracellular culture media by cells plated 24 hours prior to collection was determined using the Amplex Red Glutamic Acid/Glutamate Oxidase Assay Kit (Thermo Fisher Scientific) as described previously [31]. Data from at least 3 independent experiments was normalized to total protein or cell number.

### ROS Assays

Intracellular ROS levels were measured at 24 hours post-plating using CM-H_2_DCFDA (Thermo Fisher Scientific) as described previously [31]. Each of 3 independent experiments included a negative control of unlabeled cells to verify staining specificity. Results were normalized to cell number obtained by staining with crystal violet.

### Tumour Xenografts

Animal studies were reviewed and approved by the McMaster University Animal Research Ethics Board, complying with the Canadian Council of Animal Care guidelines. 4–6 week old female immunocompromised BALB/c *nu/nu* mice (Charles River) were implanted subcutaneously with 21-day release, 0.25 mg pellets of 17β-estradiol (Innovative Research of America) three days prior to subcutaneous injection with either MDA-MB-231 wild-type or MDA-MB-231 clone #2 (SH-4-54-resistant) cells prepared in sterile phosphate-buffered saline at 4×10^6^ cells in 100 μL per animal. The viability of cells to be injected was assessed via trypan blue exclusion staining. Pellet implants are routinely carried out to promote the growth of MDA-MB-231 cells in our xenograft models [34]. Two animals were included for each group. Tumour size was evaluated every 3 days, and animals were sacrificed on Day 25 post-injection, when tumours in the wild-type group reached a mean volume of 794 mm^3^. Tumours were excised, flash-frozen in liquid nitrogen, and stored at -80°C.

### Quantitative Real-Time PCR (qPCR)

cDNA was prepared using Superscript III and oligo dTs (Thermo Fisher Scientific) from total RNA isolated from cell pellets (Qiagen RNeasy kit). Following DNase treatment (Ambion), cNDA was used as template for qPCR on a BioRad CFX Connect Real-Time System using primers listed in Table 1 and SsoAdvanced Universal SYBR Green Supermix (BioRad). Reference gene primers to amplify either β-actin or RNA polymerase II (RPII) have been described previously [32] and are also listed in Table 2. The 2^-[Δ][Δ]Ct^ method was used to calculate relative mRNA levels [35], and results are presented as fold changes of relevant controls.

**Table 1:** Primers used for relative qPCR to validate RNA-sequencing results. Melt peaks listed were obtained on a BioRad CFX Connect Real-Time System and may vary by -/+ 1°C depending on the thermocycler.

Gene Symbol	Primer Sequence(5' to 3')	Reference Gene	Product Size (bp)	Melt Peak (°C)
***ADIPOR1***	**FOR:** TCCTGCCAGTAACAGGGAAG	*Actin*	168	86.5
	**REV:** AGGGGAAGTGTCAGTACCCG			
***AKT1***	**FOR:** GTGGACCAACGTGAGGCTC	*RPII*	132	88.0
	**REV:** GAAGGTGCGTTCGATGACAG			
***BCAR3***	**FOR:** CAGAAACATGCCGGTGAATCA	*Actin*, *RPII*	209	86.0–86.5
	**REV:** GTGGGGATTTGGAGTGGGG			
***B2M***	**FOR:** GCTATCCAGCGTACTCCAAAG	*Actin*	180	82.0–82.5
	**REV:** TCACACGGCAGGCATACT			
***CCND1***	**FOR:** CAATGACCCCGCACGATTTC	*Actin*	146	85.0
	**REV:** CATGGAGGGCGGATTGGAA			
***CCND2***	**FOR:** ACCTTCCGCAGTGCTCCTA	*Actin*	161	85.5
	**REV:** CCCAGCCAAGAAACGGTCC			
***CSF1***	**FOR:** AGACCTCGTGCCAAATTACATT	*Actin*	248	84.5–85.0
	**REV:** AGGTGTCTCATAGAAAGTTCGGA			
***FASN***	**FOR:** ACAGCGGGGAATGGGTACT	*RPII*	136	88.0
	**REV:** GACTGGTACAACGAGCGGAT			
***GSTM3***	**FOR:** TACCTCTTATGAGGAGAAACGGT	*Actin*, *RPII*	100	82.0–82.5
	**REV:** AGGAAAGTCCAGGTCTAGCTTG			
***MYC***	**FOR:** GTCAAGAGGCGAACACACAAC	*Actin*	130	84.5–85.0
	**REV:** TTGGACGGACAGGATGTATGC			
***PANX1***	**FOR:** GCTCTTTGCGATCCTCCTGTA	*Actin*	118	83.5
	**REV:** TGCACGGTTGTAAACTTTGTCAA			
***SCD***	**FOR:** ACCGCTCTTACAAAGCTCGG	*Actin*	154	84.5
	**REV:** CCACGTCGGGAATTATGAGGAT			
***SLC1A1***	**FOR:** TTCTAATGCGGATGCTGAAACT	*Actin*	146	81.0
	**REV:** CGCGCAGACCAATTTTTCC			
***SLC1A3***	**FOR:** AGCAGGGAGTCCGTAAACG	*Actin*	120	80.0–80.5
	**REV:** AGCATTCCGAAACAGGTAACTTT			
***SLC7A11 (xCT)***	**FOR:** CCTCTATTCGGACCCATTTAG	*Actin*	99	80.0–80.5
	**REV:** CTGGGTTTCTTGTCCCATATA			
***SLC25A1***	**FOR:** TTCCCCACCGAGTACGTGAA	*RPII*	147	89.5–90.0
	**REV:** GTAGAGCAGGGAGCTAAGGC			
***SOCS3***	**FOR:** CCTGCGCCTCAAGACCTTC	*Actin*	99	87.0
	**REV:** GTCACTGCGCTCCAGTAGAA			
***SUMO3***	**FOR:** GAATGACCACATCAACCTGAAGG	*RPII*	152	87.5–88.0
	**REV:** GCCCGTCGAACCTGAATCT			

**Table 2:** Reference gene primers used for relative qPCR. Melt peaks listed were obtained on a BioRad CFX Connect Real-Time System and may vary by -/+ 1°C depending on the thermocycler.

Gene Symbol	Primer Sequence (5' to 3')	Product Size (bp)	Melt Peak (°C)
***Actin***	**FOR:** CATGTACGTTGCTATCCAGGC	250	84.5–85.0
	**REV:** CTCCTTAATGTCACGCACGAT		
***RPII***	**FOR:** GGGTGCTGAGTGAGAAGGAC	138	87.0
	**REV:** AGCCATCAAAGGAGATGACG		

### Statistical Analyses

Results represent the mean ± SEM of at least three independent replicates for each experiment. Statistical differences between relevant groups were established by either t-test (denoted by stars) or 1-way ANOVA coupled with a Tukey’s post-test (denoted by different letters) using GraphPad Prism software. Results were considered significant at p <0.05. Immunoblots depict a representative image of three independent experiments.

### RNA-Sequencing Analysis

20 μl at 100 ng/μl of 3 independent biological replicate RNA samples isolated from separate passages of MDA-MB-231 wild-type and SH-4-54-resistant clone #2 cells, as well as triplicate independent biological replicates of T47D wild-type and SH-4-54-resistant clone #1 RNA samples, were submitted for RNA-sequencing (Farncombe Metagenomics Facility, McMaster University), also referred to as NextGeneration sequencing. RNA quality was validated using the RNA 6000 Nano kit and a 2100 Bioanalyzer (Agilent Technologies). A RNA library was prepared using the NEBNext Ultra Directional RNA Library Prep Kit for Illumina, with the Next Poly(A) mRNA Magnetic Isolation Module (New England Biolabs) used to enrich poly-A mRNA. Each library was sequenced on the Illumina HiSeq 1500 platform (Illumina) via HiSeq Rapid V2 chemistry with onboard cluster generation and 70 bp single-end reads. Each biological replicate was split between two lanes to mitigate lane effects, with reads being subsequently combined during analysis using the Tuxedo protocol as previously described [36]. The RNA-sequencing dataset was submitted to the National Institutes of Health (NIH) Sequence Read Archive (SRA) as SRA accession SRP078574.

The Galaxy Project, a web-based platform for biomedical data analysis, was used to perform several of the steps outlined in the Tuxedo protocol [37,38]. The FastQC tool (http://www.bioinformatics.babraham.ac.uk/projects/fastqc/) was applied to evaluate the quality of sequencing results in FASTQ format. Tophat was applied to align reads from individual biological replicates to the human GRCh38/hg38 assembly. Cufflinks created assembled transcripts, which were combined with the corresponding GRCh38/hg38 reference transcriptome annotation using Cuffmerge, creating a transcriptome assembly. Cuffdiff was used to assess transcript abundance in fragments per kilobase of transcript per million mapped reads (FPKM) and differential gene expression (DEG) by merging the transcriptome assembly with individual aligned reads created by Tophat. The false discovery rate-adjusted p-value (q-value) was set to >0.05.

The Tuxedo suite, a bioinformatics pipeline that uses FPKM data to normalize the number of reads to library size and gene length [36], was used to investigate DEGs between the two different MDA-MB-231 cells lines and between the two different T47D cell lines. It is worth noting that other software packages and pipelines for differential expression analysis that employ alternative normalization and statistical methods are available. Although no consensus on a “best practice” currently exists for RNA-sequencing, it has been noted that FPKM normalization leads to overly-conservative reports of DEG using the Tuxedo pipeline compared to other methods such as DEseq [39]. Therefore, it is possible that additional, less stringently selected DEGs could be identified in our study using alternative differential expression analysis. One advantage of the Tuxedo pipeline and FPKM data is the ability to perform further analysis of alternative splicing events, which may be of value for future investigations.

The Bioconductor package CummeRbund was used to visualize Cuffdiff output files in RStudio (version 0.99.467 44–46). This platform facilitated preparation of scatter and volcano plots for each pairwise comparison, as well as an expression level plot and heatmap comparing experimental groups. The Lander/Waterman equation (C = LN/G) was applied to calculate mean genome coverage: C denotes coverage, G corresponds to genome/transcriptome length (for RNA-sequencing), L denotes average read length, and N stands for the average number of reads [40]. The base coverage tool in Galaxy (https://toolshed.g2.bx.psu.edu/view/devteam/basecoverage/b8a9e718caa3) was used to derive the total length of annotated transcripts executed on the most recent human genome assembly (GRCh38), which was applied to calculate coverage.

DAVID, a web-based bioinformatics tool, was applied to perform ontological and KEGG pathway enrichment analyses to functionally interpret gene sets [41,42]. The list of DEGs for each pairwise comparison obtained from RNA-sequencing was imported into the “functional annotation” tool, with homo sapiens as the reference species. Enriched KEGG pathways and Gene Ontology Biological Processes (GO:BP) terms were identified with the Expression Analysis Systematic Explorer (EASE) threshold (maximum EASE score/p-value) set to a default of 0.1, which is used by DAVID to identify significant gene enrichment. Fold-enrichment representing the ratio of the proportion of input genes relative to the number of genes represented by a particular term or pathway within the reference human genome was also reported.

qPCR data was used to validate RNA-sequencing results (see section on qPCR above). For each of the 18 target genes selected for validation, pairwise comparisons were based on fold-changes calculated relative to appropriate controls (wild-type counterparts of each cell type). To determine the experimental asymmetrically distributed SEM for each mean, which is necessary for linearly representing data derived from an exponential analysis, SEMs derived from each ΔCT value were used to calculate upper and lower 2^−ΔΔCT^ values [43]. Linear regression was used to test the overall correlation between RNA-seq and qPCR results, with α set to 0.05 [44].

## Results

### Phospho-STAT3 and Phospho-STAT5 Levels Are Altered by Chronic SH-4-54 Treatment in MDA-MB-231 and T47D Treatment-Resistant Clones

**Fig 1:**
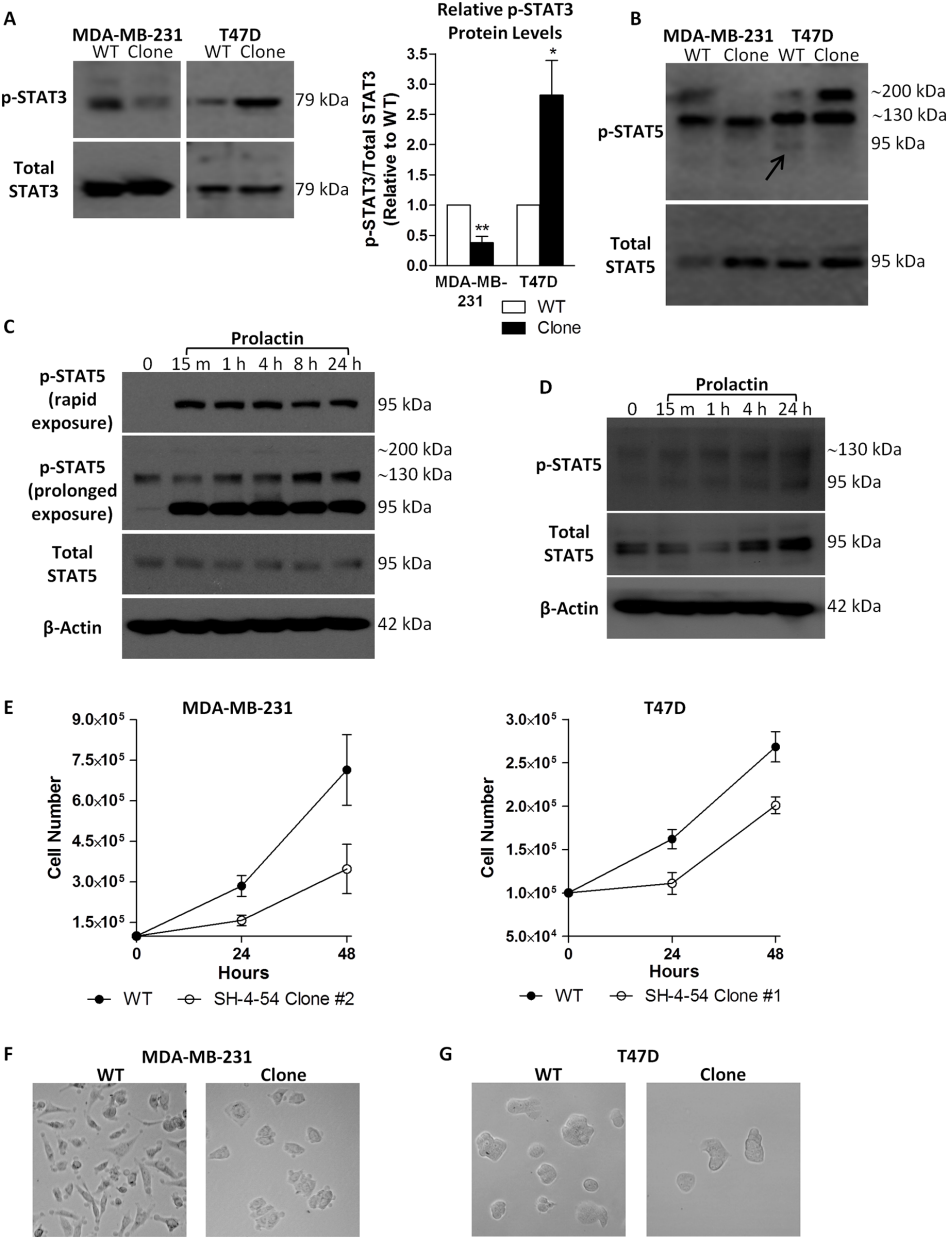
Chronic treatment of MDA-MB-231 and T47D cells with the STAT3/5 inhibitor SH-4-54, followed by clonal selection and characterization, revealed (A) different levels of phosphorylated STAT3 (p-STAT3) relative to total STAT3 in wild-type (WT) compared to clonally selected MDA-MB-231 and T47D cells, as well as (B) changes in basal levels of phosphorylated STAT5 (p-STAT5) at 95 kDa in T47D cells (indicated by the arrow). The presence of a high molecular weight p-STAT5 band at approximately 200 kDa was also inversely affected in MDA-MB-231 and T47D clonal populations relative to their WT counterparts in a cell-type dependent manner. (C) The ability of T47D cells to undergo rapid and sustained STAT5 activation in response to prolactin treatment was confirmed, with the primary band for p-STAT5 migrating at 95 kDa. (D) Increases in the canonical phosphorylation of STAT5 at 95 kDa could also be detected in MDA-MB-231 cells in response to treatment with prolactin. (E) Less cells were present over 48 hours in SH-4-54-resistant clones compared to MDA-MB-231 and T47D passage-matched WT cells. Representative bright field images (100X magnification, Leica DMIL) shows (F) the characteristic spindle-shaped morphology of WT MDA-MB-231 cells compared to marked morphological changes in SH-4-54 treatment-resistant clones, while (G) no significant changes in morphology were observed between T47D WT and SH-4-54-resistant cells. Data represent the mean of three independent experiments (±SEM) calculated relative to appropriate controls. A star (*) denotes statistically significant differences as determined by a t-test (p<0.05).

Western blotting was performed on selected MDA-MB-231 and T47D SH-4-54-resisistant clones following long-term drug treatment. Phospho-STAT3 levels were significantly lower in MDA-MB-231 resistant clones relative to wild-type MDA-MB-231 cells, with the inverse occurring in T47D resistant clones in which phospho-STAT3 levels increased by approximately 3-fold relative to their respective untreated wild-type counterpart (Fig 1A). Given that SH-4-54 inactivates both STAT3 and STAT5, its effect on phospho-STAT5 was also examined in resistant clones of both cell types. Interestingly, although the band for phospho-STAT5, which is expected to migrate at 95 kDa, was not detected after rapid exposure in wild-type MDA-MB-231 cells, two other bands at approximately 130 and 200 kDa were observed, with the latter notably absent in the SH-4-54-resistant clone (Fig 1B). A similar higher molecular weight banding pattern for phospho-STAT5 was observed in T47D cells. However, in contrast to MDA-MB-231 cells, the band at approximately 200 kDa markedly increased in overall intensity in the SH-4-54-resistant T47D clone (Fig 1B). A faint band at 95 kDa corresponding to uninduced, basal levels of phospho-STAT5 was detected in T47D wild-type cells (Fig 1B, indicated by the arrow), which was absent in its corresponding clone. This band matched the canonical migration of phospho-STAT5 obtained in response to prolactin treatment of T47D cells (Fig 1C). In addition to the 95 kDa phospho-STAT5 band induced by PRL stimulation of T47D cells, a prominent 130 kDa and very weak 200 kDa band were detected after prolonged exposure (Fig 1C), which matched the banding pattern observed in MDA-MB-231 wild-type cells treated with PRL (Fig 1D). While MDA-MB-231 cells are more resistant to canonical PRL signaling via the activation of STAT5 via JAK2 phosphorylation due to lower PRL receptor numbers compared to T47D cells [45], we have previously shown that STAT5 does become phosphorylated in response to a 100 ng/ml PRL treatment in this cell line, with maximal levels of phospho-STAT5 obtained after 24 hours [32]. Interestingly, the type of complex STAT5 banding pattern observed in the present study has been linked with SUMOylation, a particular type of post-translational modification that has been shown to significantly influence the predicted molecular weight of STAT5 during early lymphoid development [46,47]. Given that SUMOylation/de-SUMOylation is a cyclical process, we examined whether this particular STAT5 modification could be the underlying mechanism resulting in non-canonical STAT5 protein migration in breast cancer cells resistant to SH-4-54. We therefore added N-ethylmaleimide to the lysis buffer in subsequent experiments, which served to inhibit de-SUMOylation by blocking the action of SUMO proteases [33], thereby enhancing the retention of SUMOylated bands in protein extracts.

In addition to changes in the phosphorylation status of STAT3 and STAT5, a lower cell count was obtained for SH-4-54-resistant MDA-MB-231 and T47D clones over 48 hours relative to their respective untreated wild-type counterparts (Fig 1E). Lower counts were not due to changes in cell viability, given that trypan blue exclusion, which identifies non-viable cells, did not indicate any significant differences between the wild-type and SH-4-54-resistant cell lines (data not shown). Selection in SH-4-54 also significantly changed the morphology of MDA-MB-231 resistant clones compared to their wild-type counterpart (Fig 1F). No significant morphological changes were visually observed between wild-type and SH-4-54-resistant T47D cells (Fig 1G).

### xCT Expression and System x_c_^-^ Activity Is Differentially Regulated in MDA-MB-231 and T47D Clones Resistant to SH-4-54

**Fig 2:**
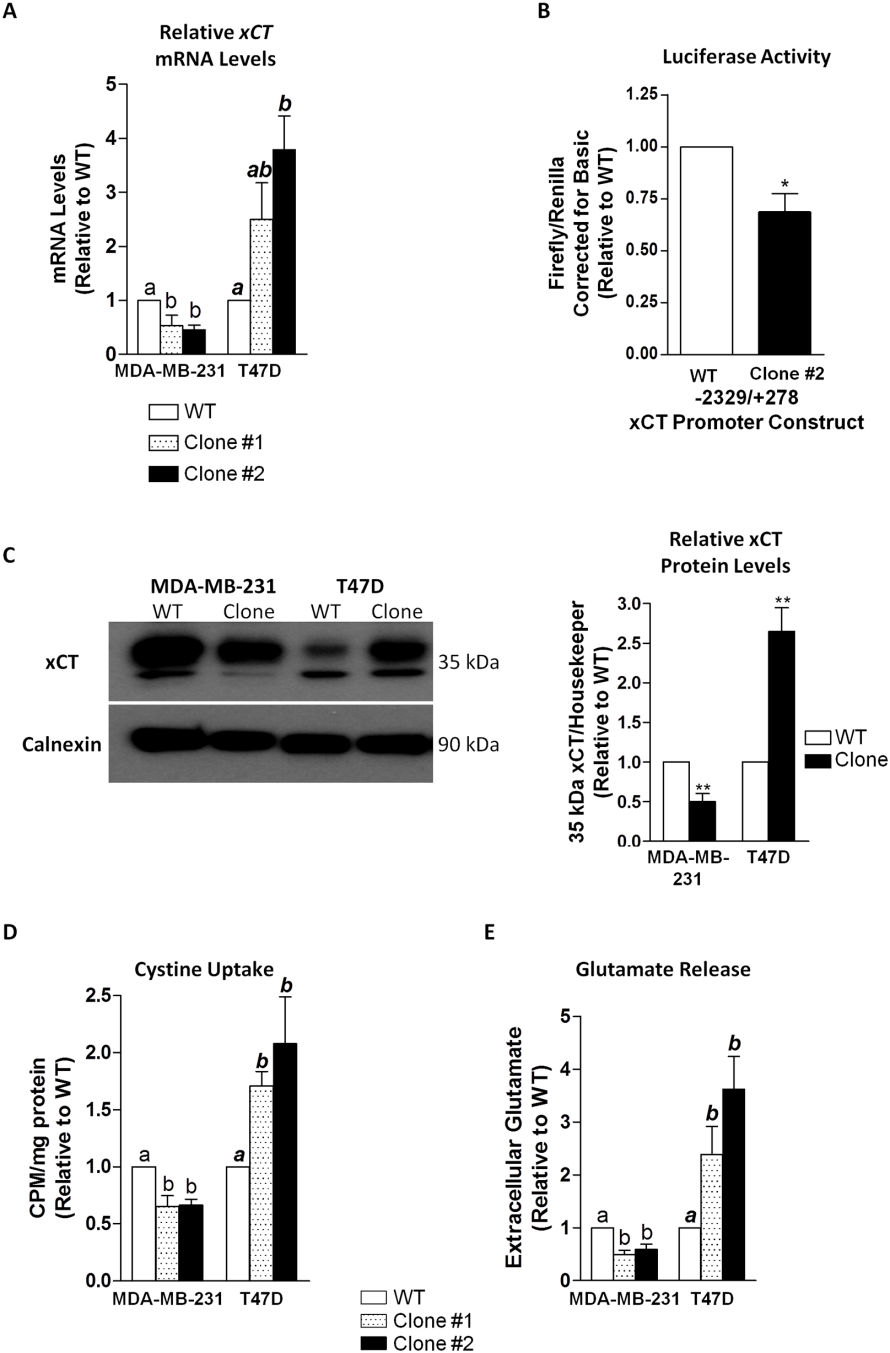
(A) xCT mRNA levels were significantly down-regulated for two different clones isolated from SH-4-54-treated MDA-MB-231 cells, while in T47D clones, mRNA levels increased significantly relative to wild-type (WT) counterparts. (B) The transcriptional activity of SH-4-54 chronically treated MDA-MB-231 clone #2 was reduced relative to luciferase activity in WT cells. (C) xCT protein levels were significantly lower in a representative MDA-MB-231 clone compared to WT cells, while chronic SH-4-54 treatment produced T47D clones in which xCT protein levels were significantly higher compared to WT T47D cells. A corresponding densitometric analysis in graphical format is presented in the left panel, corresponding to analysis of at least 3 independent blots. The activity of system x_c_^-^ was inversely affected, with relevant changes in (D) cystine uptake and (E) glutamate release demonstrating high concordance with xCT expression levels. Data represent the mean of three independent experiments (±SEM) calculated relative to appropriate controls. A star (*) denotes statistically significant differences determined by a t-test (p<0.05). Different letters a, b, or ab in panels A, C, and D correspond to statistical differences between groups (p < at least 0.05), as determined by One-way ANOVA and a Tukey’s post-test.

Two resistant clones from each cell type were selected for further analysis, denoted as clone #1 and #2. qPCR revealed that xCT mRNA levels were approximately 2-fold lower in the two MDA-MB-231 STAT3/5 inhibitor-resistant clones relative to wild-type cells, with an inverse 2 to 3-fold increase occurring in T47D clones relative to their wild-type counterpart (Fig 2A). Given that we previously showed SH-4-54 to acutely increase xCT promoter activity in MDA-MB-231 cells, we examined its chronic effects at the transcriptional level in a representative resistant clone. Relative to wild-type MDA-MB-231 cells, xCT promoter activity was significantly reduced following long-term SH-4-54 treatment (Fig 2B). Importantly, the changes in xCT mRNA levels were reflected at the protein level, densitometrically corresponding to a 2-fold decrease in MDA-MB-231 SH-4-54-resistant clones relative to wild-type cells, while xCT protein levels increased significantly by 2.5-fold in T47D clones in response to chronic SH-4-54 treatment (Fig 2C). xCT is the functional component of system x_c_^-^, and as such, changes in protein levels corresponded to significant down- or up-regulation of its activity, including cystine uptake (Fig 2D) and glutamate release (Fig 2E), in both sets of MDA-MB-231 and T47D STAT3/5 inhibitor-resistant clones, respectively.

### xCT Expression in Tumours Isolated from Murine Xenografts Remains Low in Animals Subcutaneously Injected with MDA-MB-231 Clones Resistant to Chronic SH-4-54 Treatment Relative to Wild-Type Cells

**Fig 3:**
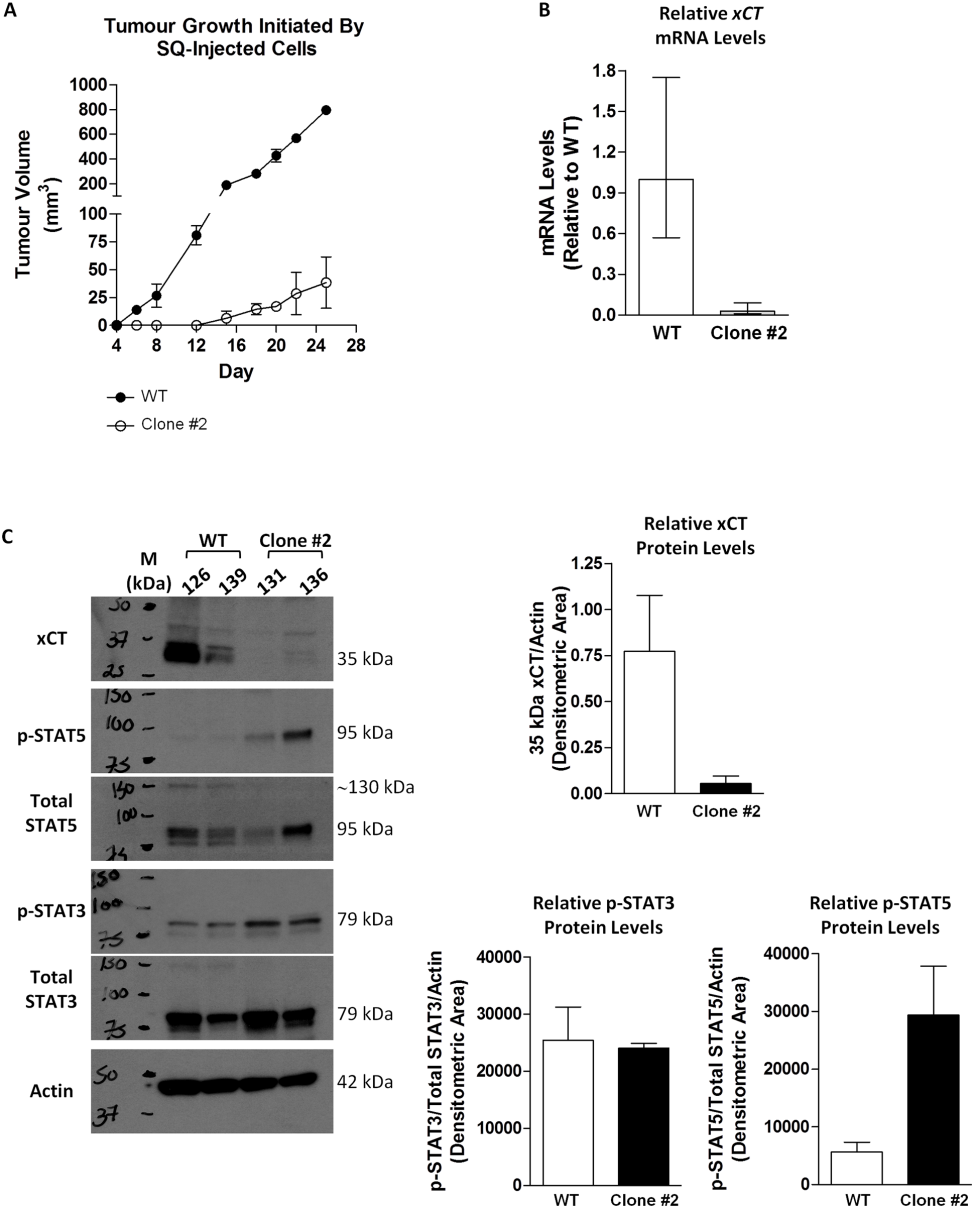
Subcutaneous injection into nude mice revealed that (A) MDA-MB-231 SH-4-54-resistant clone #2 proliferated at a slower rate than its wild-type (WT) counterpart *in vivo*. (B) qPCR demonstrated that xCT mRNA levels were lower in tumours isolated from animals injected with clone #2 relative to WT cells (2 animals per treatment group). (C) Western blot analysis of protein isolated from subcutaneous tumours derived from *in vivo* growth of the clones relative to WT-derived tumours revealed that xCT levels remained low, phospho-STAT5 (p-STAT5) levels remained high, and phospho-STAT3 (p-STAT3) levels remained unchanged in the absence of SH-4-54.

To ascertain whether chronic SH-4-54-induced repression of system x_c_^-^ is sustained *in vivo* without further drug administration, which would eliminate any effects of *in vitro* culture conditions and indicate permanent cellular changes at the gene level, we injected cells of one of the representative MDA-MB-231 resistant clones into nude mice in parallel to wild-type cells. Mean tumour growth rates (n = 2 for each cell type) revealed that wild-type-induced tumours grew at a faster rate compared to tumours initiated from SH-4-54-resistant clones (Fig 3A). qPCR demonstrated that xCT mRNA levels were reduced in tumours isolated from animals injected with SH-4-54 resistant cells compared to those injected with wild-type cells (Fig 3B). Western blot analysis of protein isolated from each tumour using standard lysis conditions confirmed changes occurring at the mRNA level, showing that xCT expression appears to be lower in animals initially injected with the SH-4-54-resistant clonal cells compared to those receiving wild-type cells (Fig 3C). In contrast, 95 kDa phospho-STAT5 levels were higher in tumours initiated from SH-4-54-resistant clones, while phospho-STAT3 levels did not change when normalized to levels of total STAT3 (Fig 3C). These results support that activated STAT5 may continue to repress xCT expression *in vivo*, and that the changes in clonally selected, treatment-resistant cancer cells are likely not transient, given that tumours were allowed to grow for 25 days post-injection in the absence of SH-4-54 prior to sacrifice and tissue collection.

### RNA-Sequencing Revealed Differentially Expressed Genes in MDA-MB-231 and T47D SH-4-54-Treated Clones Relative to Parental Wild-Type Cells

**Fig 4:**
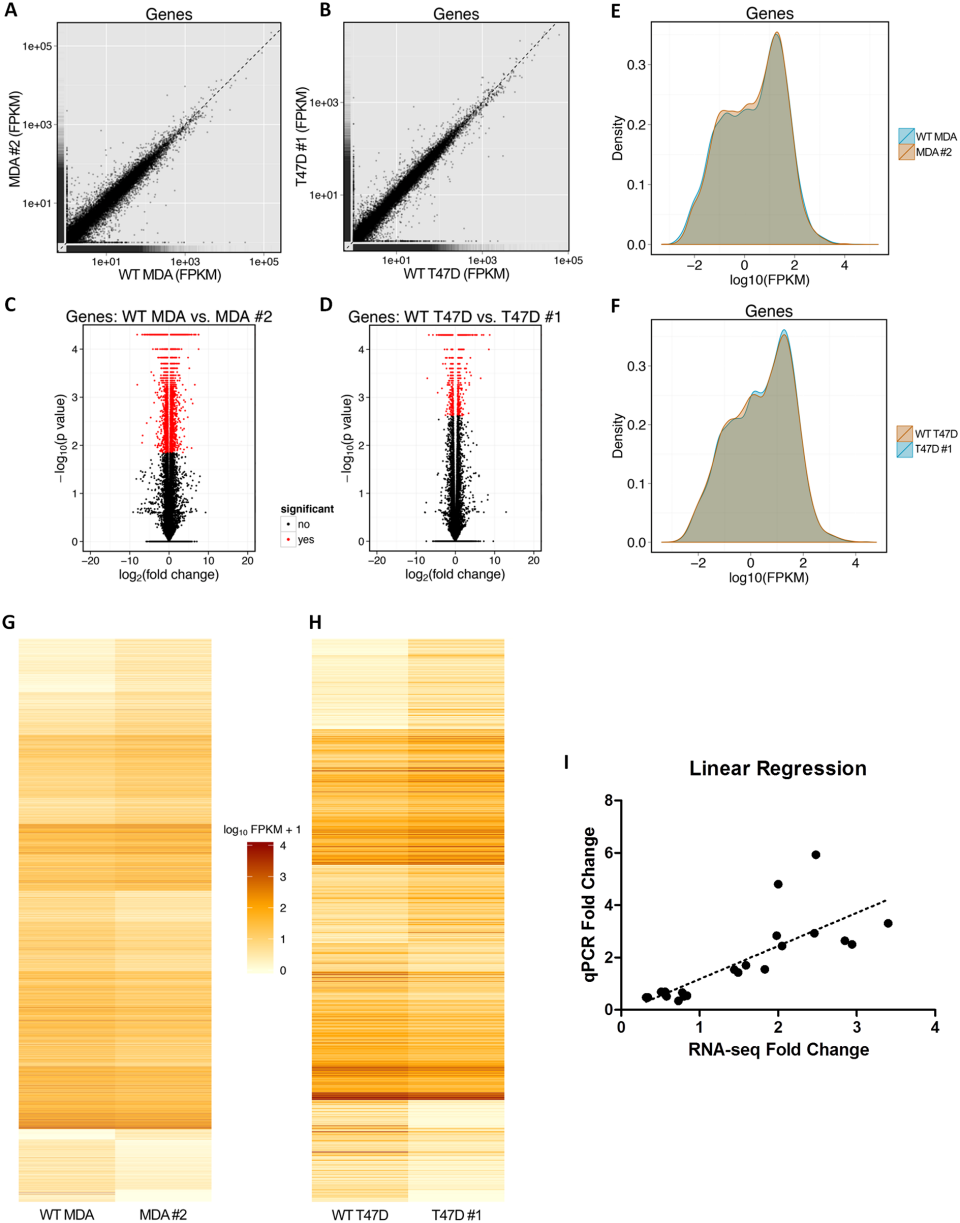
A visual summary of differential gene expression patterns derived from RNA-sequencing data. Scatter plots illustrate overall gene expression similarities and differences between (A) wild-type (WT) MDA-MB-231 (MDA) cells and MDA clone #2, and (B) WT T47D cells and T47D clone #1. Volcano plots highlight genes that were differentially expressed between (C) WT MDA cells and MDA clone #2, and (D) WT T47D cells and T47D clone #1. Density plots illustrate expression level distribution, with non-overlapping segments representing differential gene expression between (E) WT MDA cells and MDA clone #2, and (F) WT T47D cells and T47D clone #1. Heatmaps illustrate the level of gene expression [in log10(FPKM+1)] for genes that were differentially expressed between (G) WT MDA cells and MDA clone #2, and (H) WT T47D cells and T47D clone #1. FPKM: fragments per kilobase of transcript per million mapped reads. (I) Linear regression analysis of qPCR results compared with RNA-sequencing results. All pairwise comparisons and a wide range of fold-changes are represented in the analysis. Regression revealed high concordance between the two methods.

Two pairwise comparisons of DEGs were performed using RNA-sequencing: wild-type MDA-MB-231 versus MDA-MB-231 SH-4-54-resistant clone #2, and wild-type T47D versus T47D SH-4-54-resistant clone #1. Each group consisted of three independently collected biological replicates representing cells at different passages. On average, samples yielded 1347 +/- 307 Mbases (range: 692–2201 Mbases) and 19.2 +/- 4.4 million reads (range: 9.9–31.4 million reads). Coverage of the human genome was calculated to be 10.8x (70 bp × 19.2 million reads / 124.9 Mbp = 10.8), indicating that, on average, each transcript base was sequenced 10 to 11 times. Fig 4 provides a visual representation of data derived for each pairwise comparison, with scatter plots (Fig 4A and 4B) highlighting overall similarities and differences in gene expression between wild-type and SH-4-54-resistant clones within a given comparison. Volcano plots generated by plotting log2(p-value) against log2(fold-change) of individual genes (Fig 4C and 4D) highlight the DEGs in each comparison. Density plots (Fig 4E and 4F) illustrate expression level distributions, with DEGs represented by regions of non-overlapping groups. Heatmaps (Fig 4G and 4H) provide a visual representation of the expression level [in log10(FPKM+1)] for all DEGs across the two different comparisons. A total of 4227 DEGs were identified in the MDA-MB-231 wild-type versus MDA-MB-231 STAT3/5 inhibitor-resistant clone comparison, while 752 DEGs were identified in the T47D wild-type versus T47D STAT3/5 inhibitor-resistant clone comparison (S1 Table). All results obtained for GO:BP terms and KEGG pathway analysis are listed in S2 Table. A subset of 18 genes was chosen to validate fold-changes in DEGs obtained by RNA-sequencing via qPCR analysis (Table 3). Linear regression confirmed high overall concurrence between the two methods (Fig 4I).

**Table 3:** 18 DEGs identified by RNA-sequencing that were further validated by qPCR.

Cell Type	Gene	RNA-seq Fold Δ (q value)	qPCR Fold Δ (p value)
**MDA-MB-231 WT vs. Clone #2**	*ADIPOR1*	1.59 (0.00036)	1.70 (0.01)
	*AKT1*	0.78 (0.037)	0.66 (0.04)
	*BCAR3*	0.34 (0.00036)	0.48 (0.03)
	*CCDN1*	1.44 (0.00036)	1.54 (0.002)
	*CCND2*	2.94 (0.0036)	2.50 (0.00002)
	*FASN*	0.56 (0.0013)	0.59 (0.02)
	*GSTM3*	0.58 (0.00036)	0.52 (0.002)
	*MYC*	0.51 (0.00036)	0.69 (0.009)
	*PANX1*	2.05 (0.00036)	2.44 (0.0002
	*SCD*	0.32 (0.00036)	0.48 (0.03)
	*SLC1A1*	2.85 (0.00036)	2.64 (0.0007)
	*SLC1A3*	1.83 (0.0038)	1.55 (0.006)
	*SLC25A1*	0.73 (0.0007)	0.34 (0.03)
	*SLC7A11 (xCT)*	0.56 (0.00036)	0.69 (0.03)
	*SOCS3*	0.80 (0.027)	0.52 (0.0008)
	*SUMO3*	0.84 (0.040)	0.54 (0.02)
**T47D WT vs. Clone #1**	*BCAR3*	2.00 (0.015)	4.8 (0.002)
	*B2M*	1.49 (0.039)	1.43 (0.008)
	*CSF1*	2.48 (0.019)	5.93 (0.02)
	*GSTM3*	1.98 (0.0022)	2.84 (0.001)
	*MYC*	2.46 (0.0022)	2.93 (0.005)
	*SLC7A11 (xCT)*	3.40 (0.0022)	3.31 (0.02)

### STAT3 and STAT5 Target Genes Were Identified To Be Differentially Expressed in MDA-MB-231 and T47D SH-4-54-Resistant Clones Relative to Wild-Type Cells by RNA-Sequencing and qPCR

**Fig 5:**
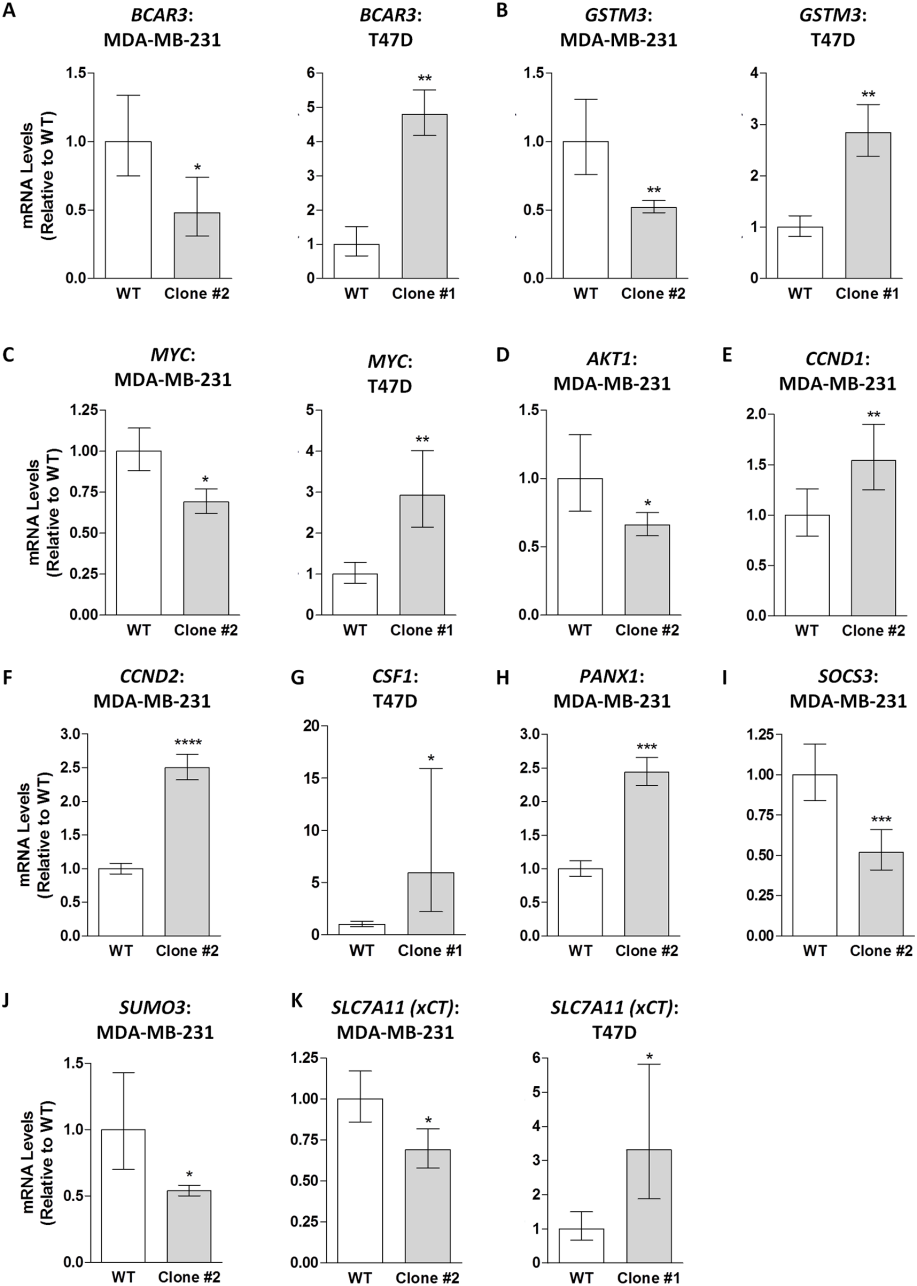
11 genes selected for graphical representation of relative qPCR fold-changes indicate significant differences, which confirmed the status of 18 total DEGs identified by RNA-sequencing (refer to Table 3). For each group, data represents the mean of 3 independent biological replicates, each analyzed in duplicate, with error bars indicating the SEM calculated using the 2^-[Δ][Δ]Ct^ method. Data represent the mean of three independent experiments (±SEM) calculated relative to appropriate controls. A star (*) denotes statistically significant differences determined using a t-test (p<0.05).

A preliminary scan of DEGs revealed several that have been reported to be directly regulated by STAT3, STAT5, or both [16,20,48–50] (Table 4). 282 of the 4227 DEGs identified in the MDA-MB-31 comparison overlapped with the T47D comparison, with 94 of these DEGs following opposite trends (if up-regulated in one comparison, the DEG was down-regulated in the other comparison, and vice versa). A list of these 94 genes is included in Table 5, with a substantial number containing putative STAT3 or STAT5 binding sites in their promoter regions based on the Weizmann Institute of Science Human Gene Database (GeneCards). Among the list in Table 5 is *MYC*, an important oncogene and known STAT3/5 target gene [15,16], which was significantly down-regulated in the MDA-MB-231 comparison while being up-regulated in the T47D comparison. Notably, *SLC7A11 (xCT)* was also on this list, being significantly down-regulated by approximately 2-fold in the MDA-MB-231 STAT3/5 inhibitor-resistant clone relative to wild-type cells while being significantly up-regulated by 3.4-fold in the T47D clone relative to wild-type cells (Tables 2 and 3). qPCR validations for several representative STAT3/5 target genes identified by RNA-sequencing are graphically represented in Fig 5, including *BCAR3* (Fig 5A), *GSTM3* (Fig 5B), *MYC* (Fig 5C), *AKT1* (Fig 5D), *CCND1* (Fig 5E), *CCND2* (Fig 5F), *CSF1* (Fig 5G), *PANX1* (Fig 5H), *SOCS3* (Fig 5I), *SUMO3* (Fig 5J), and *SLC7A11* (xCT; Fig 5K), confirming the overall specificity of results obtained by RNA-sequencing for a range of fold-changes. A complete list of 18 DEGs validated by qPCR is presented in Table 3.

**Table 4:** Subset of select genes reported by others to be regulated by STAT3 or STAT5 [16,20,48–50]. These genes were identified by RNA-sequencing to be differentially expressed in MDA-MB-231 and T47D SH-4-54-resistant clones relative to wild-type (untreated) cells.

STAT3 Target Gene		STAT5 Target Gene		Targeted by Both	
Gene Symbol	Fold Δ	Gene Symbol	Fold Δ	Gene Symbol	Fold Δ
**MDA-MB-231 WT vs. SH-4-54-resistant Clone #2**					
*AHR*	0.61	*CCND1*	1.44	*ARSD*	2.71
*AKT1*	0.78	*CCND2*	2.94	*B4GALT1*	1.34
*BIRC5*	0.67	*CHORDC1*	1.50	*BLZF1*	2.28
*CCDC25*	0.65	*GTF2H5*	1.27	*CHCHD2*	0.25
*CDC25A*	0.53	*LNPEP*	1.50	*COMMD5*	0.65
*CDKN1A*	2.62	*MAP3K5*	1.58	*DUSP10*	2.80
*CEBPD*	0.54	*MBP*	1.58	*EGR1*	3.27
*EIF5*	0.69	*SGK1*	1.36	*ELL*	0.51
*FAS*	0.51	*SLPI*	1.91	*F2RL1*	1.50
*FOS*	4.18	*SOD3*	2.33	*FST*	0.51
*HIF1A*	1.31	*SPRR2A*	#DIV/0 (↑)	*GUCY1B3*	0.44
*MMP1*	0.24	*TNFSF10*	15.68	*IFI44*	0.45
*SAA1*	22.11	*UGCG*	2.07	*JUND*	0.50
*SOCS3*	0.80			*MYC*	0.51
*TIMP1*	2.09			*NUDT12*	1.38
				*PANX1*	2.05
				*PMAIP1*	1.68
				*S100A2*	2.18
**T47D WT vs. SH-4-54-resistant Clone #1**					
*CSF1*	2.48	*SOD3*	1.93	*MYC*	2.46
*IL32*	10.57				
*JUN*	2.03				

**Table 5:** Set of 94 differentially up- or down-regulated genes identified via RNA-sequencing with opposite fold changes in MDA-MB-231 and T47D cells chronically treated with SH-4-54 relative to respective wild-type cells. Genes containing putative STAT3 or STAT5 binding elements in their promoter region are highlighted **in bold**. As *MYC* is a known STAT3/5 target gene, differentially expressed genes regulated by MYC are also indicated in bold. Genes validated by qPCR are further highlighted in grey.

Gene Symbol	MDA WT vs. Clone #2 Fold Δ	T47D WT vs. Clone #1 Fold Δ	Description
*ABCG1*	0.39	1.96	ATP-Binding Cassette, Sub-Family G (WHITE), Member 1
*AC007405*.*6*	0.57	3.93	Homo sapiens BAC clone RP11-570C16
*AC091801*.*1*	18.54	0.06	Homo sapiens chromosome UNK clone RP11-1228A3, SEQUENCING IN PROGRESS, 15 unordered pieces
*ANKMY2*	0.34	1.56	Ankyrin Repeat And MYND Domain Containing 2
***AREG***	**1.88**	**0.22**	Amphiregulin (**STAT3 promoter element**)
*ARMCX1*	0.72	1.89	Armadillo Repeat Containing, X-Linked 1 (STAT1 site in its promoter element)
*ASH1L*	2.09	0.48	Ash1 (Absent, Small, Or Homeotic)-Like (Drosophila)
*ATP11A*	1.76	0.35	ATPase, Class VI, Type 11A
***BCAR3***	**0.34**	**2.00**	Breast Cancer Anti-Estrogen Resistance 3 (**STAT5 promoter element**)
*CA12*	0.75	1.67	Carbonic Anhydrase XII
*CAMKK1*	0.59	1.59	Calcium/Calmodulin-Dependent Protein Kinase Kinase 1, Alpha
*CBX1*	0.80	1.66	Chromobox Homolog 1
*CHAC1*	0.45	2.20	ChaC Glutathione-Specific Gamma-Glutamylcyclotransferase 1
*CILP2*	2.77	0.16	Cartilage Intermediate Layer Protein 2
*CLIC3*	14.69	0.54	Chloride Intracellular Channel 3
*COL5A1*	2.92	0.24	Collagen, Type V, Alpha 1
*CYP4V2*	0.48	3.41	Cytochrome P450, Family 4, Subfamily V, Polypeptide 2
*DNMT3B*	1.33	0.63	DNA (Cytosine-5-)-Methyltransferase 3 Beta
***EPHA4***	**3.17**	**0.16**	Ephrin receptor 4A (**STAT3 promoter element**)
*EREG*	2.77	0.02	Epiregulin; Ligand of the EGF receptor/EGFR and ERBB4
*FAM149A*	0.38	2.82	Family With Sequence Similarity 149, Member A
***FAM185A***	**0.60**	**1.74**	Family With Sequence Similarity 185, Member A (**MYC-regulated**)
*FAM89A*	0.02	6.22	Family With Sequence Similarity 89, Member A
***FNBP1L***	**0.76**	**1.60**	Formin Binding Protein 1-Like (**STAT5 promoter element**)
*GALNT1*	1.29	0.62	Polypeptide N-Acetylgalactosaminyltransferase 1
*GJA1*	0.71	3.52	Gap Junction Protein, Alpha 1, 43kDa
***GNG11***	**0.71**	**1.79**	Guanine Nucleotide Binding Protein (G Protein), Gamma 11 (**STAT5 promoter element**)
*GPR141*	0.37	8.53	G Protein-Coupled Receptor 141
*GPRC5C*	0.07	2.39	G Protein-Coupled Receptor, Class C, Group 5, Member C
***GSTM3***	**0.58**	**1.98**	Glutathione S-Transferase Mu 3 (Brain) (**STAT5 promoter element**)
*HCAR1*	2.32	0.68	Hydroxycarboxylic Acid Receptor 1
*ID4*	0.26	10.77	Inhibitor Of DNA Binding 4, Dominant Negative Helix-Loop-Helix Protein
***IDH1***	**0.57**	**1.66**	Isocitrate Dehydrogenase 1 (NADP+), Soluble (**STAT5 promoter element**)
***IFI6***	**1.35**	**0.25**	Interferon, Alpha-Inducible Protein 6 (**STAT3 promoter element**)
*IL1R1*	2.92	0.61	Interleukin 1 Receptor, Type I
***INPP5E***	**1.25**	**0.66**	Inositol Polyphosphate-5-Phosphatase E (**MYC-regulated**)
*KRCC1*	0.61	1.61	Lysine-Rich Coiled-Coil 1
*KYNU*	1.35	0.60	Kynureninase
*LANCL1*	0.64	1.41	LanC Lantibiotic Synthetase Component C-Like 1 (Bacterial)
*LCP1*	4.04	0.51	Lymphocyte Cytosolic Protein 1 (L-Plastin)
*LEF1*	0.44	4.48	Lymphoid Enhancer-Binding Factor 1
*LINC00665*	1.63	0.64	Long Intergenic Non-Protein Coding RNA 665
*LINC01234*	11.64	0.18	Long Intergenic Non-Protein Coding RNA 1234
***LMO4***	**0.69**	**2.78**	LIM Domain Only 4 (**STAT3 promoter element**)
*LYAR*	1.31	0.60	Ly1 Antibody Reactive
*MAN1C1*	0.25	2.69	Mannosidase, Alpha, Class 1C, Member 1
*MDGA2*	0.00	3.78	MAM Domain Containing Glycosylphosphatidylinositol Anchor 2
*MISP*	2.68	0.64	Mitotic Spindle Positioning
***MKX***	**1.83**	**0.37**	Mohawk Homeobox (**STAT5 promoter element**)
*MMD*	0.76	1.56	Monocyte To Macrophage Differentiation-Associated (STAT1 promoter element)
*MT-CYB*	1.58	0.62	Mitochondrially Encoded Cytochrome B
***MYC***	**0.51**	**2.46**	V-Myc Avian Myelocytomatosis Viral Oncogene Homolog (**STAT3 promoter element; regulated by STAT3 and STAT5**)
***NAT8L***	**1.63**	**0.61**	N-Acetyltransferase 8-Like (GCN5-Related, Putative) (**MYC-regulated**)
*NMB*	3.52	0.33	Neuromedin B
*NT5DC4*	0.00	143.31	5'-Nucleotidase Domain Containing 4
*OAT*	0.82	1.73	Ornithine Aminotransferase
*OLFML2A*	1.48	0.50	Olfactomedin-Like 2A
*P4HA3*	35.32	0.01	Prolyl 4-Hydroxylase, Alpha Polypeptide III
*PCTP*	0.57	1.94	Phosphatidylcholine Transfer Protein
***PDE9A***	**0.00**	**2.94**	Phosphodiesterase 9A (**STAT3 & STAT5 promoter elements**)
***PDHB***	**0.76**	**1.48**	Pyruvate Dehydrogenase (Lipoamide) Beta (**STAT3 promoter element**)
*PHF11*	1.36	0.58	PHD Finger Protein 11
*PON3*	0.46	1.98	Paraoxonase 3
*PPFIA4*	3.25	0.19	Protein Tyrosine Phosphatase, Receptor Type, F Polypeptide (PTPRF), Interacting Protein (Liprin), Alpha 4 (ERα-regulated)
*PQLC3*	0.60	1.83	PQ Loop Repeat Containing 3
*PRKACB*	0.38	1.59	Protein Kinase, CAMP-Dependent, Catalytic, Beta (STAT1 promoter element)
*PSAT1*	0.55	1.76	Phosphoserine Aminotransferase 1
*PTPRS*	1.58	0.69	Protein Tyrosine Phosphatase, Receptor Type, S
*RAB39A*	2.00	0.17	Member RAS Oncogene Family
***RNF144B***	**0.48**	**2.68**	Ring Finger Protein 144B (**STAT5 promoter element**)
*RNF208*	1.62	0.63	Ring Finger Protein 208
*SARS*	0.56	1.50	Seryl-TRNA Synthetase
*SEC22B*	0.55	1.55	SEC22 Homolog B, Vesicle Trafficking Protein (Gene/Pseudogene)
*SEMA6B*	1.33	0.53	Sema Domain, Transmembrane Domain (TM), And Cytoplasmic Domain, (Semaphorin) 6B
*SLC25A24*	0.74	1.70	Solute Carrier Family 25 (Mitochondrial Carrier; Phosphate Carrier), Member 24
***SLC7A11 (xCT)***	**0.56**	**3.40**	Solute Carrier Family 7 (Anionic Amino Acid Transporter Light Chain, Xc- System), Member 11 (**STAT5 promoter element**)
*SLC9A3R1*	0.77	1.56	Solute Carrier Family 9, Subfamily A (NHE3, Cation Proton Antiporter 3), Member 3 Regulator 1
***SMAD3***	**1.55**	**0.45**	SMAD Family Member 3 (**MYC-regulated**)
*SMARCA1*	0.72	1.62	SWI/SNF Related, Matrix Associated, Actin Dependent Regulator Of Chromatin, Subfamily A, Member 1
*SNORA22*	3.81	0.22	Small Nucleolar RNA, H/ACA Box 22
*SNORA7B*	1.83	0.14	Small Nucleolar RNA, H/ACA Box 7B
*SOWAHC*	0.80	2.68	Sosondowah Ankyrin Repeat Domain Family Member C
*SSR2*	1.23	0.66	Signal Sequence Receptor, Beta (Translocon-Associated Protein Beta)
*STEAP1*	0.64	5.04	Six Transmembrane Epithelial Antigen Of The Prostate 1
*STMN3*	1.67	0.44	Stathmin-Like 3
*TENM3*	2.29	0.55	Teneurin Transmembrane Protein 3
***TGFBI***	**2.40**	**0.25**	Transforming Growth Factor, Beta-Induced, 68kDa (**STAT5 promoter element**)
*TICAM1*	1.61	0.56	Toll-Like Receptor Adaptor Molecule 1
*TMEM108*	6.73	0.40	Transmembrane Protein 108
*TRIB3*	0.59	1.52	Tribbles Pseudokinase 3
*TSKU*	0.70	2.08	Tsukushi, Small Leucine Rich Proteoglycan
***TSPAN13***	**0.32**	**1.92**	Tetraspanin 13 (**MYC-regulated**)
*TSPAN33*	0.27	2.25	Tetraspanin 33 (STAT1 promoter element)
*TUB*	0.06	2.38	Tubby Bipartite Transcription Factor
***VAT1***	**0.79**	**1.55**	Vesicle Amine Transport 1 (**MYC-regulated**)

### STAT5 Is De-SUMOylated in SH-4-54-Resistant MDA-MB-231 Clones

**Fig 6:**
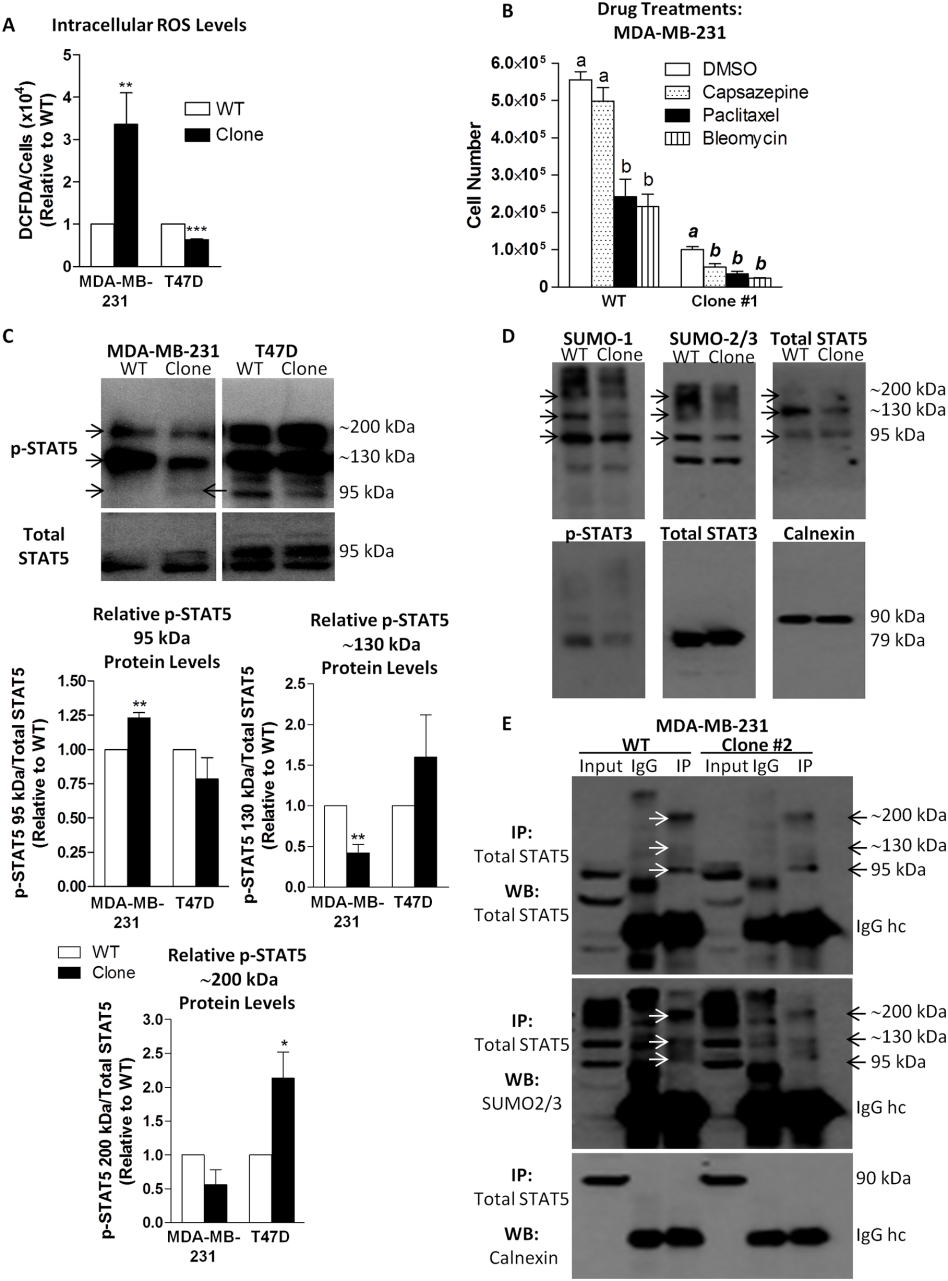
(A) Levels of intracellular ROS were significantly higher in MDA-MB-231 SH-4-54-resistant clones relative to wild-type (WT) cells, while in clones derived from resistant T47D cells, ROS levels were significantly lower than their WT counterpart. (B) Treatment with capsazepine, paclitaxel, or bleomycin resulted in lower cell counts of MDA-MB-231 clones compared to vehicle (DMSO), whereas the number of WT MDA-MB-231 cells was not affected by treatment with capsazepine. (C) Western blotting revealed that levels of phospho-STAT5 (p-STAT5) at 95 kDa increased significantly in MDA-MB-321 clones relative to their WT counterpart (indicated by the arrow), with an overall decrease in the intensity of bands at approximately 130 and 200 kDa, while in T47D clones, the opposite occurred. (D) Western blots of lysates derived from MDA-MB-231 WT and clones probed with SUMO-1, SUMO-2/3, and total STAT5 antibodies revealed similar banding patterns, with common bands observed at approximately 95, 130, and 200 kDa. Lower overall band intensities were observed in SH-4-54-resistant clones compared to their WT counterpart, with decreased levels of phospho-STAT3. (E) A representative set of IPs confirmed that SUMO-2/3 co-migrated with total STAT5 in MDA-MB-231 cells, and that levels of SUMOylated STAT5 were lower in SH-4-54-resistant clones than in WT cells (compare lanes 3 and 6). Data represent the mean of three independent experiments (±SEM) calculated relative to appropriate controls. A star (*) denotes statistically significant differences determined using a t-test (p<0.05).

In response to changes in xCT expression, intracellular ROS levels could also change due to the resulting effects on system x_c_^-^ activity. DCFDA analysis revealed that in MDA-MB-231 SH-4-54-resistant clones, ROS levels were approximately 3-fold higher than in their wild-type counterpart, with the inverse occurring in T47D cells (Fig 6A). Given that inhibition of STAT3 activity has been linked with sensitizing resistant cells to chemotherapy [51], we examined the effects of paclitaxel, a chemotherapeutic drug commonly used to treat breast cancer, as well as bleomycin and capsazepine, two ROS-inducing agents, on the proliferation of MDA-MB-231 cells. While low doses of paclitaxel and bleomycin significantly reduced the number of both wild-type and SH-4-54-resistant cells at 72 hours post-treatment, the effects of capsazepine were only observed in the latter group (Fig 6B), suggesting that this agent likely has a distinct mechanism of action.

In response to changes in ROS levels, cells may also undergo changes in SUMOylation/de-SUMOylation cycles [52,53]. It has been reported that STAT5 may be regulated through SUMOylation, which is reflected by incremental 40 kDa changes in its protein migration pattern relative to the canonical 95 kDa band [46,47]. In Fig 1B, we observed that detection of phospho-STAT5 by standard Western blotting produced an unexpected migration pattern in both MDA-MB-231 and T47D cells that included bands at approximately 130 and 200 kDa in addition to the expected band at 95 kDa. This was similar to banding patterns associated with STAT5 SUMOylation reported by others [47]. Using lysis buffer conditions that included N-ethylmaleimide, which blocks the action of de-SUMOylating enzymes [33], we confirmed the presence of these bands in human breast cancer cells, demonstrating that levels of 95 kDa phospho-STAT5 increased modestly, but significantly, in MDA-MB-231 SH-4-54-resistant clones while decreasing in T47D SH-4-54 resistant clones relative to their respective wild-type counterparts (Fig 6C). Furthermore, the intensity of bands at approximately 130 and 200 kDa decreased by 2-fold in MDA-MB-231 clones relative to wild-type cells while increasing in T47D SH-4-54-resistant cells (Fig 6C). Under these specific lysis conditions, anti-SUMO-1 and anti-SUMO-2/3 antibodies detected bands that migrated in a pattern similar to the 95, 130, and 200 kDa bands detected for total STAT5 protein in MDA-MB-231 cells (Fig 6D), suggesting that changes in the SUMOylation of total STAT5 may affect STAT5 phosphorylation, which may be related to phospho-STAT3 inactivation in SH-4-54-resistant cells derived from this cell line. Consistent with the notion of SUMO-2/3 modulating STAT5 activation, RNA-sequencing revealed that SUMO-3 mRNA levels were significantly down-regulated in MDA-MB-231 SH-4-54-resistant clones relative to wild-type cells (S1 Table), which was validated by qPCR (Fig 5J). In order to definitively establish that STAT5 is indeed differentially SUMOylated, immunoprecipitations were carried out, demonstrating co-migration of total STAT5 and SUMO-2/3 (Fig 6E). Furthermore, STAT5 was less SUMOylated in MDA-MB-231 STAT3/5 inhibitor-resistant clones than in wild-type cells (Fig 6E, compare lanes 3 and 6), with no changes in the overall intensity of the IgG heavy chain across groups.

## Discussion

System x_c_^-^ provides an important adaptive mechanism that helps stabilize the cellular consequences of altered metabolism in aggressive cancer cells by clearing excess ROS, ensuring long-term survival. STAT3 and STAT5 control the expression of numerous genes related to cancer cell metabolism, proliferation, and survival, integrating signals from diverse extracellular stimuli including cytokines, growth factors, hormones, and ROS. Various cytokines and growth factors induce ROS production [54,55], and in turn, ROS inactivate tyrosine phosphatases [56], thereby potentially up-regulating the phosphorylation/activation of STAT3, STAT5, and other important signaling molecules [57]. It has also been shown that oxidative stress and ROS production may interfere with the canonical JAK/STAT signaling pathway [58,59], and that STAT3 and STAT5 may have mitochondrial functions [24]. STAT3 and STAT5 are therefore intricately and complexly involved in oxidative metabolism in cancer cells (reviewed in [23]), although literature conflicts regarding their regulation by ROS as well as their influence on ROS production. In an extension of our previous investigation that examined the effects of acute STAT3/5 inhibition on system x_c_^-^ in human breast cancer cells, representative subtypes of resistant breast cancer cells clonally selected through long-term treatment with the novel STAT3/5 inhibitor, SH-4-54, were assessed for changes in xCT expression and system x_c_^-^ activity relative to untreated wild-type cells using functional and RNA-sequencing-based analyses.

Triple-negative, metastatic MDA-MB-231 cells were chosen as representatives of basal B (claudin-low, ER-negative, PR-negative, HER2-negative) human breast cancer cells that have an intermediate response to chemotherapy [60] and express high levels of xCT [61]. This cell line also constitutively expresses high levels of phospho-STAT3 and is more resistant to stimulus-induced STAT5 activation. In contrast, T47D cells represent luminal A (ERα-positive, PR-positive, HER2-negative) cells that are largely responsive to both endocrine and chemotherapy [60]. T47D cells have low levels of constitutive phospho-STAT3 and phospho-STAT5, but respond rapidly and robustly to stimuli that induce both STAT3 and STAT5-mediated signaling, such as prolactin [21]. Both represent cell lines that do not express the HER2/neu receptor, and therefore cannot be clinically targeted using trastuzumab, a monoclonal antibody that interferes with the HER2/neu receptor used to treat HER2-positive metastatic breast cancer [62]. Application of these particular cell types provided a starting point to launch our investigation into potential treatment resistance potent STAT3/5 inhibitors. Chronically blocking STAT3/5 phosphorylation and subsequent DNA binding with SH-4-54 resulted in the isolation of resistant MDA-MB-231 clones that proliferated more slowly and displayed down-regulated xCT mRNA and protein levels accompanied by a decrease in system x_c_^-^ activity. While the SH-4-54-resistant clones derived from wild-type T47D cells also grew more slowly over a 48 hour period relative to their wild-type counterparts, xCT expression was up-regulated concomitant with functional increases in cystine uptake and glutamate release. Importantly, differential changes in *SLC7A11* (*xCT*) mRNA levels were validated by RNA-sequencing, further confirming that STAT3/5 signaling is central to the transcriptional regulation of xCT in human breast cancer cells. In addition to these changes, the morphology of MDA-MB-231 cells was affected by chronic SH-4-54 treatment, with cells adopting a more rounded phenotype relative to the typical spindle shape associated with wild-type cells. The morphology of SH-4-54-resistant T47D clones did not change significantly, with cells retaining the cluster-like formation typical of wild-type T47D cells. Classification of human breast cancer cell lines has established an association between biological characteristics, including morphology and invasiveness, and transcriptionally defined subtypes [63]. While basal B cells are more “mesenchymal-like” and appear less differentiated, luminal cells form tight cell-cell junctions, appearing more homogenous and, therefore, more differentiated [63]. Based on their altered morphology, it is tempting to speculate that the MDA-MB-231 SH-4-54-resistant cells are more luminal than basal-like, which remains to be experimentally evaluated.

We previously showed that SH-4-54, which is able to block both phospho-STAT3 and phospho-STAT5, induces rapid ROS production in human breast cancer cells (within 4 hours of treatment), and that increases in SH-4-54-induced xCT promoter activity may be abolished by pre-treating MDA-MB-231 cells with N-acetylcysteine. These acute changes (within 24 hours) are likely due to the effect of SH-4-54 blocking phospho-STAT3, given that WP1066, a small molecule inhibitor know to block JAK2/STAT3 signaling by degrading JAK2 protein, produced identical effects [31]. We therefore speculated that short-term changes in xCT promoter activity are likely due to increased NRF-2 pathway activation in response to increased levels of intracellular ROS, resuling in up-regulated xCT transcriptional activity. Interestingly, while a commercially available STAT5 inhibitor that blocks phospho-STAT5 without targeting the activation of STAT3 also upregulated xCT promoter activity, xCT mRNA levels, and levels of xCT protein, it did not acutely alter cystine uptake or ROS production, suggesting that STAT5 could be acting as a direct transcriptional repressor in an ROS-independent manner [31]. This supported our previous finding that the STAT5 inhibitor completely abrogated STAT5A binding to the xCT promoter region containing a GAS/STAT site [31]. In light of results produced in the current investigation following chronic STAT3/5 inhibition with SH-4-54 in MDA-MB-231 cells, we provide further evidence that the effect of STAT5 on xCT expression occurs at the gene level, inhibiting xCT transcriptional activity and expression. As a consequence, system x_c_^-^ activity is also decreased in selected MDA-MB-231 clones, leading to higher ROS levels. Interestingly, chronic treatment of T47D cells with SH-4-54 produced clones in which phospho-STAT3 levels increased, culminating in up-regulated xCT expression, increased function of system x_c_^-^, and lower levels of intracellular ROS, which is consistent with increased cystine uptake countering any potential increases in ROS by promoting GSH synthesis. These results suggest that STAT3 and STAT5 have distinct functions in regulating the overall expression of xCT in breast cancer cells.

In addition to confirming changes in xCT expression at the mRNA level, RNA-sequencing revealed changes in the expression levels of numerous known STAT3 and STAT5 target genes. For example, *MYC*, a well-known hallmark of aggressive cancers, was validated by qPCR to follow the same pattern of differential gene expression as *xCT* in MDA-MB-231 and T47D SH-4-54-resistant clones relative to their respective wild-type counterparts. Another noteworthy putative STAT5 target gene, *BCAR3* (breast cancer anti-estrogen resistance 3), which encodes a SRC homology 2 domain by which it may direct cellular signaling to increase cell motility and estrogen-independent proliferation in human breast cancer cells [64], was confirmed by qPCR to be significantly down-regulated by approximately 2-fold in MDA-MB-231 SH-4-54-resistant clones while being significantly up-regulated by at least 2-fold in T47D clones. Specific types of breast cancers initially depend on estrogen for tumour growth and disease progression, largely responding well to anti-estrogen therapies including tamoxifen. However, these breast cancers often become treatment-resistant, and *BCAR3* was identified as being involved in the development of anti-estrogen resistance [65]. The STAT3/5 status may induce changes in estrogen-responsiveness and the ERα-status of breast cancer cells, which is supported by the finding that STAT5 may regulate ERα [66]. The altered genotype of T47D SH-4-54-resistant clones reported here may therefore contribute to an estrogen-independent phenotype, warranting further investigation.

In cancer cells, mitochondria produce ROS as a consequence of up-regulated metabolic activity, mediate apoptosis, and are also targeted by ROS-induced damage we have previously shown that STAT3 is able to bind to the xCT promoter [31], demonstrating that inhibition of STAT3 activation may directly affect system x_c_^-^ to affect cellular redox balance. However, chronic suppression of STAT3 activity in SH-4-54-resistant MDA-MB-231 clones could also be further disrupting ROS levels through mitochondrial effects, including STAT3-mediated regulation of the electron transport chain (ETC) (reviewed in [31]). One of several protective means by which mitochondrial quality may be maintained in oxidatively stressed cells, represented by the STAT3/5-resistant MDA-MB-231 clones characterized in the current investigation, is through the autophagy/lysosome pathway (reviewed in [67]). Lysosomal responses to ROS that are generated from perturbations in cellular metabolism, including changes in the activity of system x_c_^-^, are closely linked with redox homeostasis. Interestingly, the lysosome was an enriched KEGG pathway identified through further analysis of the MDA-MB-231 RNA-sequencing comparison. Numerous important genes associated with lysosomal activity, most notably cathepsin B, D, F, K, S, and Z (*CTSB* to *CTSZ*), which play an important role in cellular protein turnover [68], were significantly up-regulated in SH-4-54-resistant MDA-MB-231 cells (S1 and S2 Tables). In keeping with the autophagy/lysosome pathway playing a potential pro-survival role in STAT3/5 inhibitor-resistant MDA-MB-231 clones, which could equip them to “manage” increased levels of ROS as a consequence of permanent xCT down-regulation, KEGG pathway analysis of the MDA-MB-231 comparison identified changes in the apoptosis pathway (S2 Table), which included significant down-regulation of *FAS* (FAS cell surface death receptor), *FADD* (FAS-associated via death domain), *CYCS* (cytochrome C), *DFFA* (DNA fragmentation factor alpha), and *DFFB* (DNA fragmentation factor beta; caspase-activated DNase) (S1 Table). From the RNA-sequencing results that indicate an overall down-regulation of pro-apoptostic genes, which will need to be functionally confirmed in future experiments, we speculate that MDA-MB-231 SH-4-54-resistant clones have “adapted”, to a certain extent, to higher levels of intracellular ROS. This is supported by the observation that, while MDA-MB-231 SH-4-54-resistant clones proliferated more slowly than their wild-type counterpart, their overall survival was not significantly affected.

**Fig 7:**
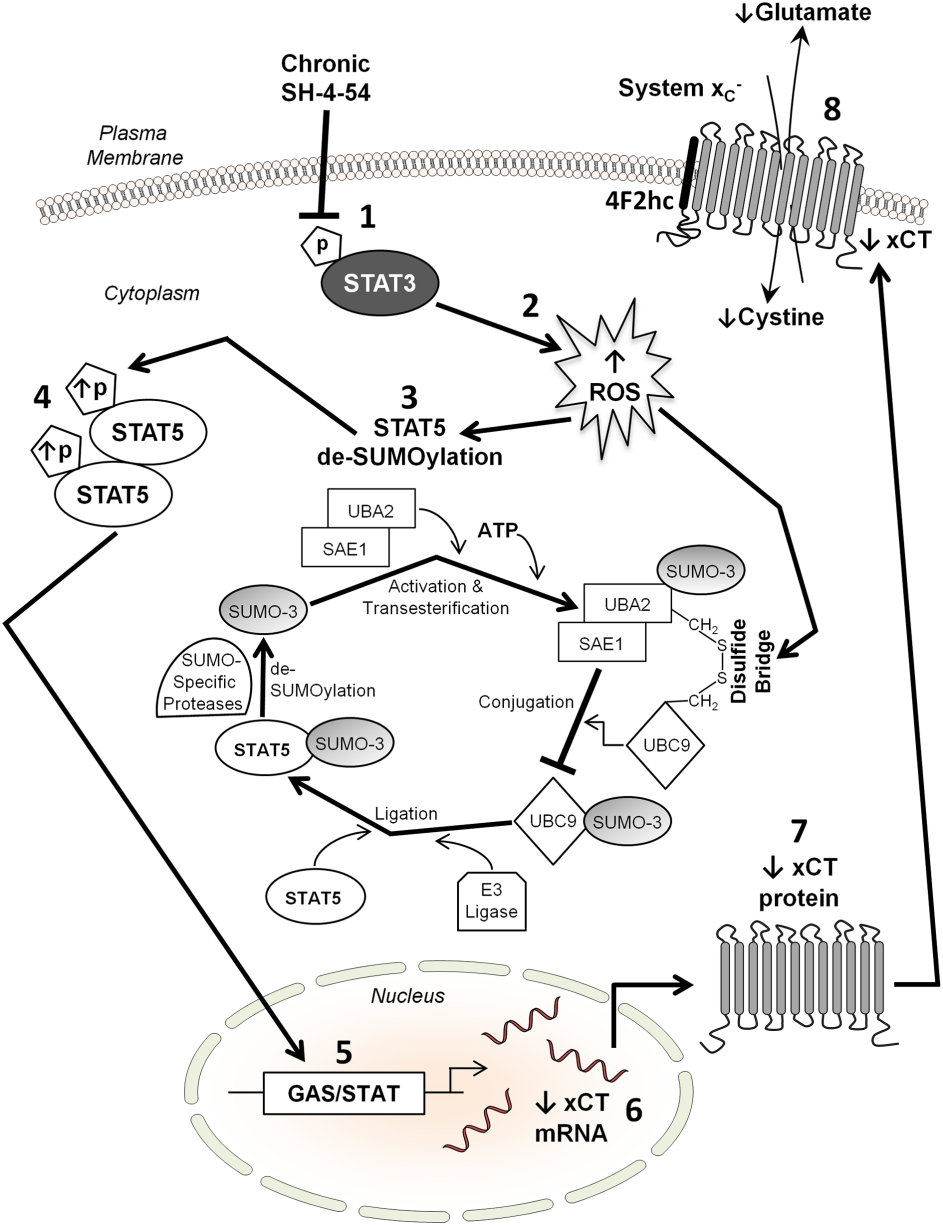
Proposed mechanism by which *xCT* is down-regulated in response to chronic SH-4-54 treatment, resulting in resistant clones of a subtype of aggressive, triple-negative human breast cancers cells (represented in the current study by MDA-MB-231 cells): (1) inhibiting constitutive STAT3 phosphorylation via continuous SH-4-54 administration (2) increases the level of intracellular ROS, resulting in (3) de-SUMOylation of STAT5 (details shown), which (4) enables STAT5 to be phosphorylated. Activated STAT5 translocates to the nucleus, (5) where it functions as a transcriptional repressor by binding to a GAS/STAT site in the *xCT* promoter, thereby (6) reducing *xCT* mRNA and (7) xCT protein levels. This ultimately destabilizes the cellular redox balance by (8) limiting the activity of system x_c_^-^ at the plasma membrane, reducing the import of cystine concomitant with the export of glutamate.

A key finding that emerged from our study was that STAT5 SUMOylation/de-SUMOylation mediates, at least in part, differential STAT5 activation in MDA-MB-231 and T47D SH-4-54-resistant cells. SUMOylation/de-SUMOylation is a post-translational process that is influenced by fluctuating ROS levels (reviewed in [52,53]). Indeed, a shift toward de-SUMOylation may be a central mechanism by which cells attempt to regain homeostasis in response to oxidative stress [52]. SUMOylation changes the properties of the protein that is targeted by SUMO moieties, in turn affecting diverse cellular processes including DNA replication and repair, mitosis, signal transduction, nuclear transport, as well as the regulation of transcription [69]. The STAT5 protein harbors several SUMOylation sites at its C-terminal end [46]. Importantly, STAT5 activation has been shown to be influenced by SUMOylation during early lymphoid development, with SUMO-STAT5 antagonizing STAT5 tyrosine phosphorylation, thereby impairing STAT5 nuclear signaling [46,47]. We have shown that in MDA-MB-231 SH-4-54-resistant clones, increased STAT5 phosphorylation is linked with de-SUMOylation, most likely via the action of two nearly identical proteins collectively referred to as SUMO-2/3 [53]. In support of this notion, RNA-sequencing validated by qPCR showed that SUMO-3, a ubiquitin-like protein covalently attached as a monomer or polymer to lysine residues on target proteins [53], was significantly down-regulated in SH-4-54-resistant MDA-MB-231 clones. Covalent substrate attachment of SUMO-3 to a target protein requires several steps, including prior ATP-dependent activation and transesterification of the SUMO-specific, heterodimeric E1 ligase complex SAE1-UBA2 (SAE2) [70] and subsequent linkage to the E2 ligase UBC9, which is encoded by the *UBE2I* gene [71]. Others have shown that UBC9 plays a key role in STAT5 activation during lymphopoiesis [47]. Importantly, RNA-sequencing revealed that *UBE2I* expression was also significantly down-regulated in MDA-MB-231 SH-4-54-resistant clones (S1 Table). Following conjugation of the SUMO moiety to UBC9, an E3 ligase transfers it onto the substrate protein, and SUMO-specific proteases then de-SUMOylate the target, which can then be activated and undergo nuclear translocation as part of a cycle that is summarized in Fig 7. While most SUMOylated proteins undergo cyclical SUMO conjugation/de-conjugation, oxidative stress may influence this process (reviewed in [53]). Indeed, ROS have been shown to inactivate the SUMO cycle by inducing the formation of a disulfide bridge between UBA2 and UBC9, resulting in overall net substrate protein de-SUMOylation [72]. This fits well with our finding that in MDA-MB-231 SH-4-54 resistant clones, in which ROS levels are higher than their wild-type counterpart, STAT5 is de-SUMOylated, while the inverse occurs in T47D resistant clones that display lower ROS levels than their parental cells. Numerous other SUMO protein substrates have been identified in mammalian cells, including, for example, DNMT3A/B, FAS, HIF1α, JUN, PCNA, and p53 (reviewed in [53]), with several of these targets also being identified in our RNA-sequencing analysis (S1 Table). It is therefore possible that changes in the expression of these and other cellular factors is an indirect effect of chronic STAT3/5 inhibition in MDA-MB-231 cells that involves ROS-mediated de-SUMOylation, which remains to be explored.

From our findings, it appears that selectively further destabilizing the cellular redox status may be critical to produce clinically meaningful outcomes linked to chronic treatment with SH-4-54 and other potent STAT3/5 inhibitors. Others have shown that a low dose of capsazepine generates significant levels of ROS, thereby sensitizing colorectal cancer cells to apoptosis [73]. Interestingly, capsazepine effectively reduced the number of SH-4-54-resistant MDA-MB-231 clones without affecting their wild-type counterpart. In contrast, treatment with bleomycin, as well as the chemotherapeutic agent paclitaxel, which interferes with the breakdown of microtubules during mitosis and is used to treat breast carcinomas [74], significantly reduced cell numbers in both resistant and wild-type MDA-MB-231 cells. Relevantly, it has been shown in acute myeloid leukemia cells that chemotherapeutic drugs ROS-dependently inhibit the SUMO-conjugating enzyme UBC9, and that pro-oxidants or inhibition of the SUMO pathway by anacardic acid are able to initiate apoptosis in chemoresistant patient samples and leukemic stem cells [75]. Inhibiting STAT3/5 activity with SH-4-54 in conjunction with further raising intracellular ROS levels beyond the cellular detoxification capacity while simultaneously affecting the SUMO pathway may be a viable therapeutic strategy to target treatment-resistant cancer cells. In support of this notion, inhibiting constitutively activated STAT3 resensitizes drug-resistant lymphomas and myelomas to cisplatin, fludarabine, adriamycin, and vinblastine-induced apoptosis [51], and pairing the cyclooxygenase inhibitor ibuprofen with pharmacological system x_c_^-^ inhibition using sulfasalazine synergistically improves its antitumor efficacy in a murine sarcoma model [76]. However, understanding the underlying mechanism of drug action may be a critical component in selecting effective therapeutic combination treatments. Capsazepine may have a unique mechanism of action distinct from bleomycin, which produces ROS during its induction of DNA strand breaks [77]. Interestingly, we speculate that the action of bleomycin may not be a relevant mode of inducing ROS, as expression of glutathione peroxidase 7 (*GPX7*), which plays a role in protecting esophageal epithelial cells against oxidative DNA damage and double-strand breaks [78], was identified by RNA-sequencing to be significantly up-regulated in MDA-MB-231 SH-4-54-resistant clones relative to wild-type cells (S1 Table). We have previously shown that at 25 μM, capsazepine effectively inhibits glutamate release through system x_c_^-^ in MDA-MB-231 cells [79], and as chronic SH-4-54 treatment does not eliminate *xCT* expression entirely, capsazepine may therefore be blocking any remaining system x_c_^-^ activity, which would explain our findings.

Our study may have a significant impact on determining the therapeutic efficacy of novel STAT3/5 small molecule inhibitors such as SH-4-54 aimed at treating breast cancer and other aggressive cancers in which the ying-yang effects of STAT proteins play a central role, such as leukemias. SH-4-54 is highly effective at inducing cell death in glioblastomas [10] and in MDA-MB-231 and T47D cells (based on our observations during the selection of treatment-resistant clones). Nevertheless, treatment-resistant cells do emerge, as is the case with most drug regimens aimed at eradicating cancer cells. We have shown that in representative SH-4-54-resistant subtypes of breast cancer cells, shifts in cellular redox balance and changes in the SUMOylation profile may prove therapeutically beneficial, as they may be more vulnerable to further induction of ROS production. This is particularly relevant given that the SUMO cycle is dysregulated in multiple myeloma, has been associated with an adverse outcome in cancer patients [80], and inhibition of a SUMOylation-dependent transcriptional program induces death of *MYC*-over-expressing cancer cells [81]. Consistent with reports by others [75], our study demonstrates that cancer cells exhibiting a de-SUMOylated profile produce smaller tumours in murine xenografts. Importantly, these tumours also express lower levels of xCT coupled with higher levels of phosophorylated STAT5 compared to wild-type MDA-MB-231 cells in which STAT5 is SUMOylated, even in the absence of continued SH-4-54 treatment.

## Conclusion

The current study has identified that inhibition of constitutive STAT3 phosphorylation with a novel and potent STAT3/5 inhibitor, SH-4-54, induces ROS, thereby affecting the SUMO pathway by favoring de-SUMOylation and subsequent phosphorylation of STAT5. Activated STAT5 is then able to serve as a transcriptional repressor at the *xCT* gene locus, reducing xCT expression and destabilizing one of the important redox balancing mechanisms of an aggressive triple-negative breast cancer subtype by limiting cystine uptake through system x_c_^-^ (Fig 7). This ultimately renders relevant target cells more susceptible to oxidative stress. In contrast, chronic SH-4-54 treatment of representative ERα-positive cells that initially respond to STAT5 signals results in STAT3 activation and up-regulation of xCT, potentially leading to a more aggressive cancer subtype.

## Supporting Information

S1 Table List of all DEGs identified in the MDA-MB-231 and T47D RNA-sequencing comparisons (wild-type vs. SH-4-54-resistant clones). (XLSX)

S2 Table Enriched KEGG pathways and Gene Ontology Biological Processes (GO:BP) terms identified via DAVID in the MDA-MB-231 and T47D RNA-sequencing comparisons (wild-type vs. SH-4-54-resistant clones). (XLSX)
